# Association of *RYR2* Mutation With Tumor Mutation Burden, Prognosis, and Antitumor Immunity in Patients With Esophageal Adenocarcinoma

**DOI:** 10.3389/fgene.2021.669694

**Published:** 2021-05-17

**Authors:** Zaoqu Liu, Long Liu, Dechao Jiao, Chunguang Guo, Libo Wang, Zhaonan Li, Zhenqiang Sun, Yanan Zhao, Xinwei Han

**Affiliations:** ^1^Department of Interventional Radiology, The First Affiliated Hospital of Zhengzhou University, Zhengzhou, China; ^2^Interventional Institute of Zhengzhou University, Zhengzhou, China; ^3^Interventional Treatment and Clinical Research Center of Henan Province, Zhengzhou, China; ^4^Department of Hepatobiliary and Pancreatic Surgery, The First Affiliated Hospital of Zhengzhou University, Zhengzhou, China; ^5^Department of Endovascular Surgery, The First Affiliated Hospital of Zhengzhou University, Zhengzhou, China; ^6^Department of Colorectal Surgery, The First Affiliated Hospital of Zhengzhou University, Zhengzhou, China

**Keywords:** esophageal adenocarcinoma, mutation, RYR2, prognosis, immunotherapy

## Abstract

**Background**: Esophageal adenocarcinoma (EAC) remains a leading cause of cancer-related deaths worldwide and demonstrates a predominant rising incidence in Western countries. Recently, immunotherapy has dramatically changed the landscape of treatment for many advanced cancers, with the benefit in EAC thus far been limited to a small fraction of patients.

**Methods**: Using somatic mutation data of The Cancer Genome Atlas (TCGA) and the International Cancer Genome Consortium, we delineated the somatic mutation landscape of EAC patients from US and England. Based on the expression data of TCGA cohort, multiple bioinformatics algorithms were utilized to perform function annotation, immune cell infiltration analysis, and immunotherapy response assessment.

**Results**: We found that *RYR2* was a common frequently mutated gene in both cohorts, and patients with *RYR2* mutation suggested higher tumor mutation burden (TMB), better prognosis, and superior expression of immune checkpoints. Moreover, *RYR2* mutation upregulated the signaling pathways implicated in immune response and enhanced antitumor immunity in EAC. Multiple bioinformatics algorithms for assessing immunotherapy response demonstrated that patients with *RYR2* mutation might benefit more from immunotherapy. In order to provide additional reference for antitumor therapy of different *RYR2* status, we identified nine latent antitumor drugs associated with *RYR2* status in EAC.

**Conclusion**: This study reveals a novel gene whose mutation could be served as a potential biomarker for prognosis, TMB, and immunotherapy of EAC patients.

## Introduction

Esophageal cancer is the eighth most prevalent malignancy and the sixth leading cause of cancer-related mortality worldwide. The predominant subtype in Western countries is esophageal adenocarcinoma (EAC), which demonstrated a predominant rising incidence in the last 40 years (Rustgi and El-Serag, 2014; Siegel et al., 2019). Gastroesophageal reflux disease is a strong risk factor for EAC, wherein the normal lower esophageal squamous epithelium is replaced with an intestinal-type columnar mucosa (Barrett’s esophagus), which can give rise to EAC (Rustgi and El-Serag, 2014). Despite advances in multi-modality treatment including endoscopic treatment, surgery, chemotherapy, and radiotherapy, the overall survival (OS) of EAC patients remains unsatisfactory (Messager et al., 2016). Thus, novel therapeutic strategies are urgently needed, especially for patients’ refractory to conventional therapies.

In recent years, immunotherapy has made tremendous progress and provided encouraging evidence (Ganesh et al., 2019). As a typical representative of immunotherapy, immune checkpoint inhibitors (ICIs) aim to help the immune system recognize and attack cancer cells by acting on the primary targets including programmed death-ligand 1 (*PD-L1*), programmed death 1 (*PD-1*), and cytotoxic T-lymphocyte-associated protein 4 (*CTLA-4*; Mahoney et al., 2015). Response to ICIs has been shown to be more effective in cancers with a high tumor mutation burden (TMB), and EAC is one example of a cancer type with a high TMB. Recent clinical trials including NCT01928394, NCT01943461, and NCT01772004 demonstrated that *PD-L1* expression in EAC is predictive of immunotherapy response (Humphries et al., 2020). Nevertheless, accumulating evidence showed that *PD-L1* alone might not be sufficient to predict the immunotherapy response due to the fact that only a minority of patients benefit. Consequently, considering the expensive cost and adverse reaction of immunotherapy, it is essential to explore novel biomarkers for effective immunotherapy management in patients with EAC.

Somatic mutations are also predictors of immunotherapy (IJsselsteijn et al., 2019). For instance, *POLE* mutation in colorectal cancer tended to respond favorably to immunotherapy (IJsselsteijn et al., 2019), mutations in *SERPINB3* and *SERPINB4* were associated with immunotherapy response in two independent cohorts of patients with melanoma (Riaz et al., 2016), and TMB had also been considered as a predictive biomarker of multiple solid tumors (Goodman et al., 2017). The genetic landscape of EAC has been well described. The Cancer Genome Atlas (TCGA) and the International Cancer Genome Consortium (ICGC) have provided large-scale comprehensive genomic characterization of EAC. Numerous efforts have been made to identify tumor drivers such as *TP53*, *SMAD4*, *ARID1A*, *SMARCA4*, and *PIK3CA*, which play essential roles in the development, progression, drug sensitivity and resistance as well as prognosis of EAC (Dulak et al., 2013; Frankell et al., 2019). We hypothesize that there are some potential frequently mutated genes (FMGs) that also could identify patients who responded to immunotherapy. Unlike traditional immunotherapeutic biomarkers such as *PD-1*/*PD-L1*, *CTLA-4*, and TMB, binary gene mutation data do not require a cutoff value to stratify patients, which conveniently promotes clinical translation.

In the present study, we delineated somatic mutations in EAC patients from US and England using TCGA and ICGC datasets. Then, the common FMGs of two cohorts were identified, and we further explored the relationship of these FMGs with TMB and OS. Ultimately, *RYR2* mutation was found to be significantly associated with TMB and OS and indicated an “immune-hot” phenotype and better immunotherapy response. The finding that will emerge from this study might identify a novel biomarker for prognosis, TMB, and immunotherapy of EAC patients.

## Materials and Methods

### Data Acquisition

Somatic gene mutation data for American EAC patients (*n* = 87) and British EAC patients (*n* = 409) were, respectively, derived from TCGA^1^ and ICGC.^2^ “Level 3” transcriptome profile [RNA-Seq fragments per kilobase per million reads (FPKM) value] and clinical information were also retrieved. The FPKM value was converted to transcripts per kilobase million value. Since RNA-seq data are often heavily right-skewed in the linear scale, a further log-2 transformation was performed. Patients were excluded if they (1) lacked somatic mutation data, (2) did not have prognostic information, and (3) received neo-adjuvant therapy.

### Delineate the Mutation Landscape

Mutation MAF files encompassing somatic alterations for American EAC patients were processed *via* VarScan pipeline. TSV files, including somatic alterations for British EAC patients, were processed with R software. The maftool package was further utilized to visualize the mutation waterfall plots. In each independent cohort, the genes with the top 30 mutation frequency were defined as frequently mutated genes (FMGs).

### Calculate the Tumor Mutation Burden for Each Patient

TMB was defined as the number of somatic, coding, indels mutations, and base substitutions per megabase of genome examined. All base substitutions and indels in the coding region of the targeted genes were counted. Silent mutations failing to contribute to an amino acid change were not counted. The *tmb()* function of “maftools” R package was applied to calculate the TMB of each sample (Mayakonda et al., 2018).

### Functional Enrichment and Immune Infiltration Analysis

To explore the potential molecular mechanisms significantly associated with *RYR2* mutation, gene set enrichment analysis (GSEA) algorithm was performed to identify dramatically enriched terms related to the Kyoto Encyclopedia of Genes and Genomes (KEGG) pathway and the biological process of gene ontology (GO). Permutations were set to 1,000 to obtain a normalized enrichment score (NES). Gene sets with false discovery rate (FDR) <0.01 were considered to be significantly enriched.

Single sample gene set enrichment analysis (ssGSEA) was applied to quantify the relative abundance of 28 immune cells in the tumor microenvironment of EAC. The gene set for marking each cell was obtained from the research of Charoentong et al. (2017), which stored various human immune cell subtypes including activated CD8^+^ T cell, activated dendritic cell, natural killer T cell, macrophage, etc. Besides this, in order to ensure the rationality and robustness of the ssGSEA results, we applied two other different algorithms to further validate. The first one was CIBERSORT, a deconvolution algorithm that took a set of reference gene expression values as a minimum representation of each cell type and, based on these values, used support vector regression to estimate the proportion of 22 immune cell types (Newman et al., 2015). The other was ESTIMATE algorithm, inferring the fraction of stromal and immune cells in EAC samples and generating two scores, including the immune and stromal scores (Yoshihara et al., 2013).

### Immunotherapy Assessments

T cell-inflamed gene expression profile (GEP) as proposed by Ayers et al. (2017) was used to predict the clinical response to *PD-1* blockade. The GEP was composed of 18 inflammatory genes associated with antigen presentation, chemokine expression, cytotoxic activity, and adaptive immune resistance. The Tumor Immune Dysfunction and Exclusion (TIDE) algorithm was employed to predict the immunotherapy response of each patient (Jiang et al., 2018). The TIDE algorithm was a computational method to model two primary mechanisms of tumor immune evasion: the induction of T cell dysfunction in tumors with high infiltration of cytotoxic T lymphocytes (CTL) and the prevention of T cell infiltration in tumors with low CTL level. Next, Subclass Mapping (SubMap) method was utilized to evaluate the expression similarity between the two *RYR2* phenotypes and the patients with a different immunotherapy response (Hoshida et al., 2007). SubMap employs GSEA algorithm to deduce the extent of commonality of the two groups. An adjusted value of *p* < 0.05 suggests the significant similarity between two groups.

### Estimation of Clinical Chemotherapeutic Response

To evaluate the drug response, we retrieved the imputed response to 138 anticancer drugs in EAC patients from a previous study (Geeleher et al., 2017). Drug sensitivity was quantified by half-maximal inhibitory concentration (IC50); a low IC50 indicates a sensitive response. We planned to identify antitumor drugs with specific sensitivity to different *RYR2* status: (1) because the IC50 value of each drug was not normally distributed (Kolmogorov-Smirnov normality test, *p* < 0.05), Wilcoxon rank-sum test was utilized; (2) considering the large number of drugs, we adopted an FDR < 0.05 as the screening criteria. FDR was obtained by Benjamini-Hochberg (BH) multiple test correction; and (3) for each drug of interest, if Wilcoxon rank-sum test FDR < 0.05 and the sensitivity of one phenotype was significantly higher than that of another phenotype, it was considered that the drug had specific sensitivity to this phenotype.

### Statistical Analysis

The gene mutation waterfall plot was visualized with “maftools” R package, and the co-occurrence or mutual exclusivity of gene mutations was evaluated by Fisher exact test. Kolmogorov-Smirnov normality test value of *p* TMB, IC50, immune cell infiltration abundance, and immune checkpoints (ICP) expression were all less than 0.05. Thus, the comparisons of two groups were conducted by Wilcoxon rank-sum test. Chi-square test or Fisher exact test was used to compare categorical variables. GSEA was performed by “clusterProfiler” R package (Yu et al., 2012). Kaplan-Meier method was applied to generate the survival curves for prognosis analyses, and log-rank test was used to define the significance of differences. The hazard ratios (HRs) for variables were calculated by univariate Cox regression analyses, and multivariate Cox regression was employed to ascertain independent prognostic factors. All statistical value of *p* were two-sided, and *p* < 0.05 was deemed as statistically significant. FDR was obtained by BH multiple test correction. All data processing, statistical analysis, and plotting were conducted in R 4.0.2 software.

## Results

### Landscape of Somatic Mutations in EAC

**Figure 1:**
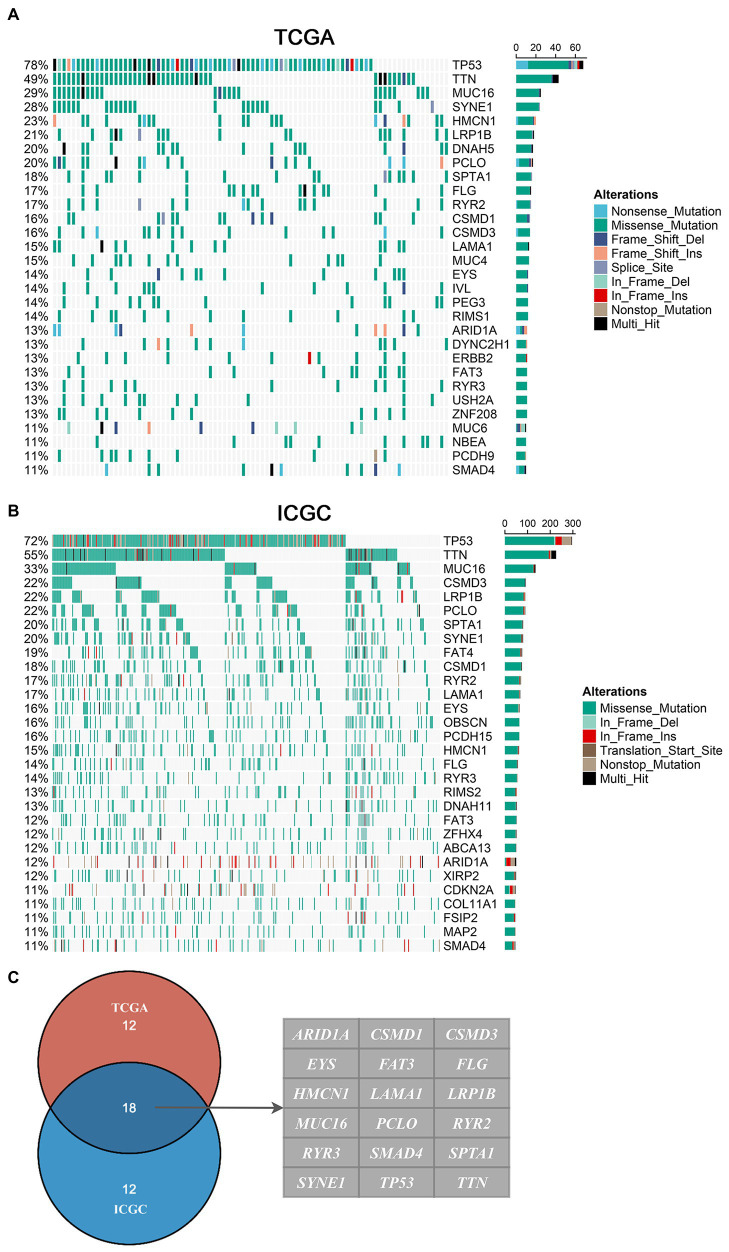
Landscapes of frequently mutated genes (FMGs) in esophageal adenocarcinoma (EAC). **(A,B)** Oncoplot depicts the FMGs of EAC in The Cancer Genome Atlas (TCGA; **A**) and International Cancer Genome Consortium (ICGC; **B**) cohorts. The left panel shows mutation rate, and genes are ordered by their mutation frequencies. The right panel presents different mutation types. **(C)** Venn diagram of FMGs covered by both TCGA and ICGC cohorts.

In the American cohort, the mutation landscape of 87 patients was summarized in this study. A total of 12,587 somatic mutations were detected, including 7,107 mutational genes and 280 genes with a mutation frequency of more than 5%. In the British cohort, the mutation landscape of 409 patients was summarized in this study. A total of 64,940 somatic mutations were detected, including 14,772 mutational genes and 254 genes with a mutation frequency of more than 5%. Consistently, missense mutation occupied the dominant fraction, and C > T displayed the highest frequency, followed by T > C and C > A. We defined 30 FMGs in American EAC patients from the TCGA cohort, and the top five FMGs were *TP53* (78%), *TTN* (49%), *MUC16* (29%), *SYNE1* (28%), and *HMCN1* (23%; Figure 1A). Meanwhile, we also defined 30 FMGs in British EAC patients from the ICGC cohort, and the top five FMGs were *TP53* (72%), *TTN* (55%), *MUC16* (33%), *CSMD3* (22%), and *LRP1B* (22%; Figure 1B). Intriguingly, some FMGs were shared by both American and British patients, including *ARID1A*, *CSMD1*, *CSMD3*, *EYS*, *FAT3*, *FLG*, *HMCN1*, *LAMA1*, *LRP1B*, *MUC16*, *PCLO*, *RYR2*, *RYR3*, *SMAD4*, *SPTA1*, *SYNE1*, *TP53*, and *TTN* (Figure 1C). Then, we focused on these common FMGs in a subsequent analysis.

### *RYR2* Mutation Was Associated With TMB and Prognosis

**Figure 2:**
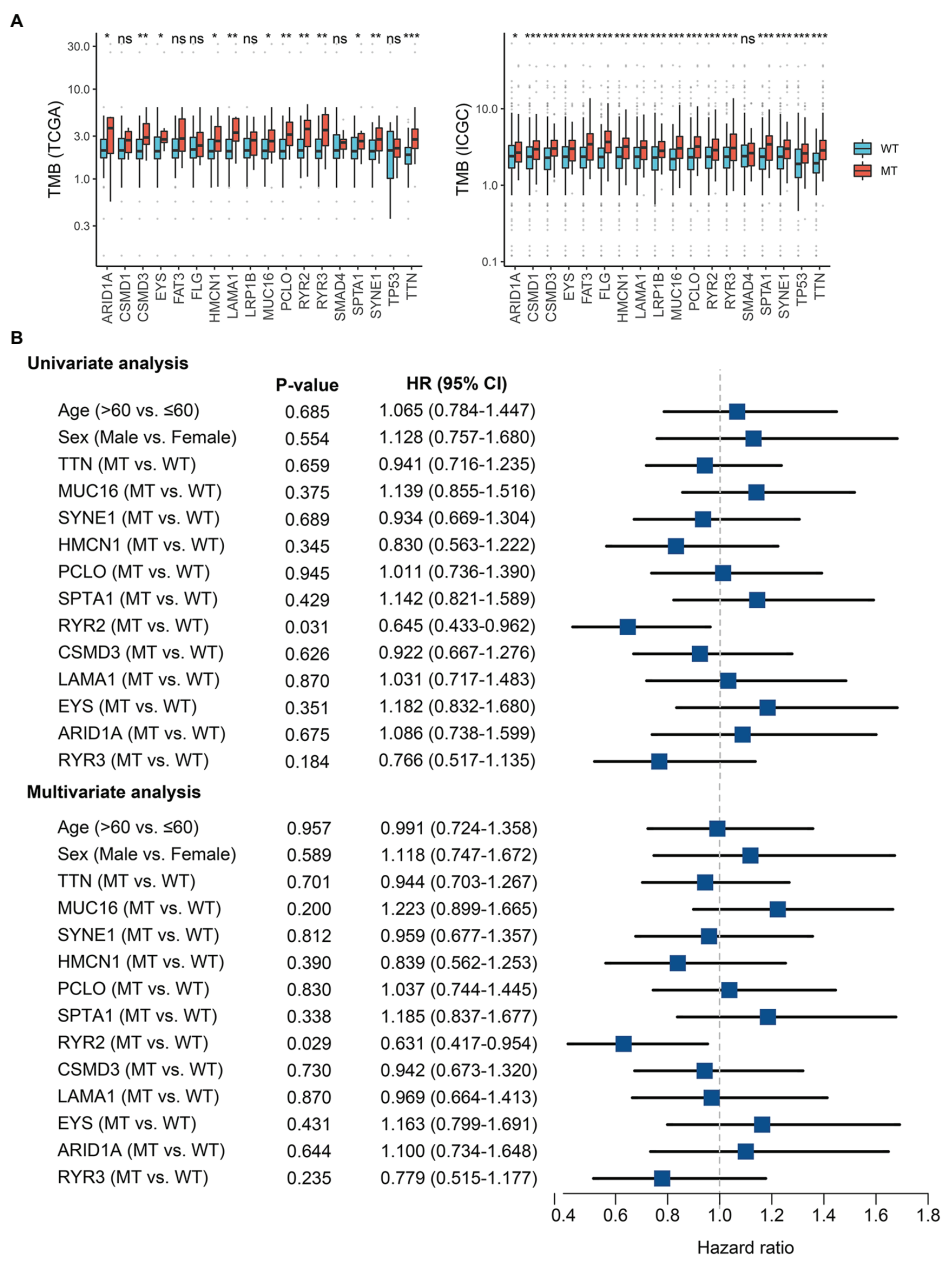
*RYR2* mutation was associated with tumor mutation burden (TMB) and clinical prognosis. **(A)** Most gene mutations are associated with a higher TMB. ns, *p* ≥ 0.05; ^*^p < 0.05; ^**^p < 0.01; ^***^p < 0.001. **(B)** Univariate and multivariate Cox regression analysis. WT, wild type and MT, mutant type.

The TMB in the TCGA cohort ranged from 0.04 to 31.70/MB, with a median of 2.1/MB; the TMB in the ICGC cohort ranged from 0.02 to 36.94/MB, with a median of 2.3/MB. Among common FMGs, patients with mutations in *ARID1A*, *CSMD3*, *EYS*, *HMCN1*, *LAMA1*, *MUC16*, *PCLO*, *RYR2*, *RYR3*, *SPTA1*, *SYNE1*, and *TTN* possessed a dramatically higher TMB in both TCGA and ICGC cohorts (Figure 2A). Previous research has demonstrated that a higher TMB suggested a favorable prognosis in multiple cancers (Liu et al., 2019). In EAC, we further explore the prognosis role of TMB. As shown in Supplementary Figure S1A, patients with high TMB displayed an improved prognosis in the TCGA American cohort (*p* = 0.094; HR = 0.588, 0.314–0.998), and similar results were demonstrated in the ICGC British cohort (*p* = 0.022, HR = 0.723, 0.547–0.956). Subsequently, survival analysis was further performed to identify whether these FMGs associated with increased TMB were also related to the OS of patients with EAC. As shown in Supplementary Figure S1B, patients with *RYR2* mutation had a significantly longer OS (*p* < 0.05). Univariate Cox analysis revealed that the HR of *RYR2* mutation was 0.645 (95% CI: 0.433–0.962; *p* < 0.05; Figure 2B). After taking into account age, gender, and mutation of other FMGs, *RYR2* mutation still remained statistically significant (*p* < 0.05), suggesting that *RYR2* mutation was an independent protective factor of prognosis in EAC (Figure 2B). The two cohorts shared the same mutation frequency of *RYR2* (17%), with 15/89 altered samples in TCGA American cohort and 70/409 altered samples in ICGC British cohort.

### *RYR2* Mutation Promoted Antitumor Immunity in EAC

**Figure 3:**
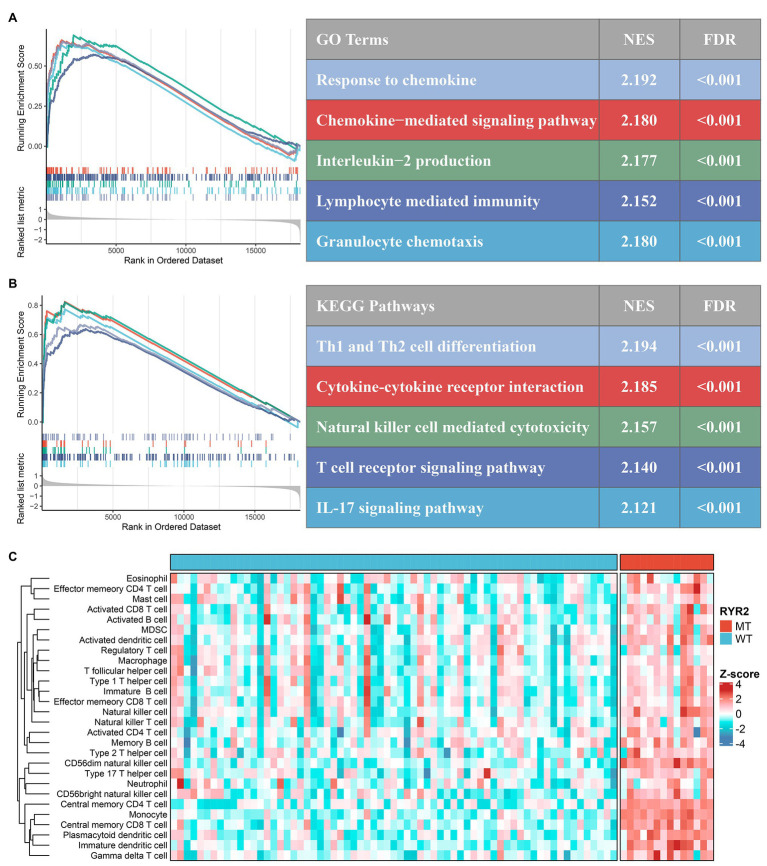
Functional and immune infiltration analysis. **(A)** Significantly enriched Gene Ontology terms associated with *RYR2* mutation. **(B)** Significantly enriched Kyoto Encyclopedia of Genes and Genomes pathways associated with *RYR2* mutation. **(C)** Assessment of infiltration abundance of 28 immune cells in patients with and without *RYR2* mutation.

According to GSEA analysis, we found plenty of immune-related GO terms that were enriched in *RYR2* mutation phenotype, such as “response to chemokine” (NES = 2.192, FDR < 0.001), “chemokine-mediated signaling pathway” (NES = 2.180, FDR < 0.001), “interleukin-2 production” (NES = 2.177, FDR < 0.001), “lymphocyte-mediated immunity” (NES = 2.152, FDR < 0.001), and “granulocyte chemotaxis” (NES = 2.180, FDR < 0.001; Figure 3A). *RYR2* mutation was also significantly associated with abundant immune-related KEGG pathways, such as “Th1 and Th2 cell differentiation” (NES = 2.194, FDR < 0.001), “cytokine-cytokine receptor interaction” (NES = 2.185, FDR < 0.001), “natural killer cell-mediated cytotoxicity” (NES = 2.157, FDR < 0.001), “T cell receptor signaling pathway” (NES = 2.140, FDR < 0.001), and “IL-17 signaling pathway” (NES = 2.121, FDR < 0.001; Figure 3B). In addition, we further applied the ssGSEA algorithm to evaluate the relative infiltration abundance of 28 immune cell types. Consistent with the above-mentioned results, the abundance of most immune cell infiltrations in patients with *RYR2* mutation was significantly higher than in patients without *RYR2* mutation (*p* < 0.05; Figure 3C and Supplementary Figure S2A). The CIBERSORT results shared a consistent immune infiltration pattern with the ssGSEA method in EAC (Supplementary Figure S2B). The ESTIMATE algorithm further demonstrated that patients with *RYR2* mutation possessed a stronger level of immune signature compared with patients without *RYR2* mutation (Supplementary Figure S2C). Overall, these results indicated that *RYR2* mutation might promote antitumor immunity in EAC, which had important implications for immunotherapy.

### *RYR2* Mutation Suggested Better Immunotherapy Response

**Figure 4:**
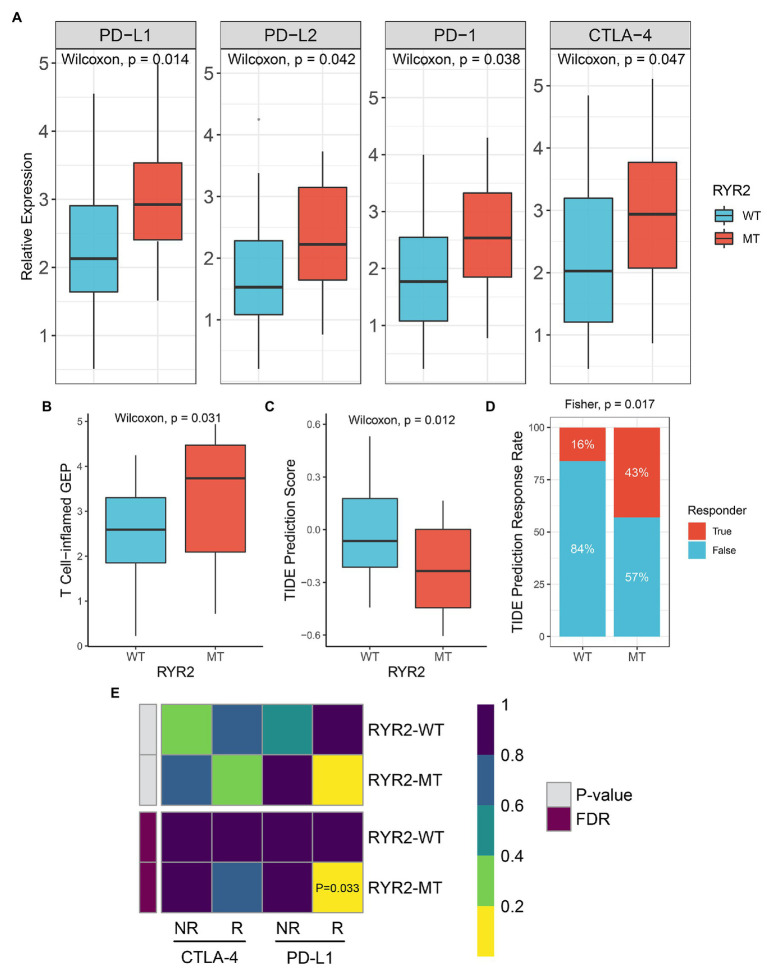
*RYR2* mutation suggested better immunotherapy response. **(A)** Expression distribution of *PD-L1*, *PD-L2*, *PD-1*, and *CTLA-4* between patients with and without *RYR2* mutation. **(B,C)** Distribution of T cell-inflamed gene expression profile **(B)** and Tumor Immune Dysfunction and Exclusion (TIDE) prediction score **(C)** between patients with and without *RYR2* mutation. **(D)** Distribution of immunotherapy responders predicted by TIDE algorithm between patients with and without *RYR2* mutation. **(E)** SubMap algorithm evaluated the expression similarity between the two phenotypes and the patients with a different immunotherapy response.

Patients with *RYR2* mutation had a higher expression level of *PD-L1*, *PD-L2*, *PD-1*, and *CTLA-4* than patients without *RYR2* mutation (Figure 4A). The T cell-inflamed GEP algorithm was utilized and a superior inflamed score was found in the *RYR2* mutation phenotype (Figure 4B). We further applied the TIDE algorithm to assess the TIDE prediction score of each patient and whether a patient would respond to immunotherapy. The TIDE prediction score was lower in the *RYR2* mutation phenotype (Figure 4C). In addition, the proportion of responders to immunotherapy in patients with *RYR2* mutation was higher relative to the patients without *RYR2* mutation (mutant type vs. wild type: 43 vs. 16%; Figure 4D). The SubMap analysis also revealed the dramatic expression similarity between the *RYR2* mutation phenotype and patients with anti-PD-L1 therapy (FDR < 0.05; Figure 4E). These results indicated that *RYR2* mutation suggested better immunotherapy response.

### Identification of Potential Antitumor Drugs Associated With *RYR2* Status

**Figure 5:**
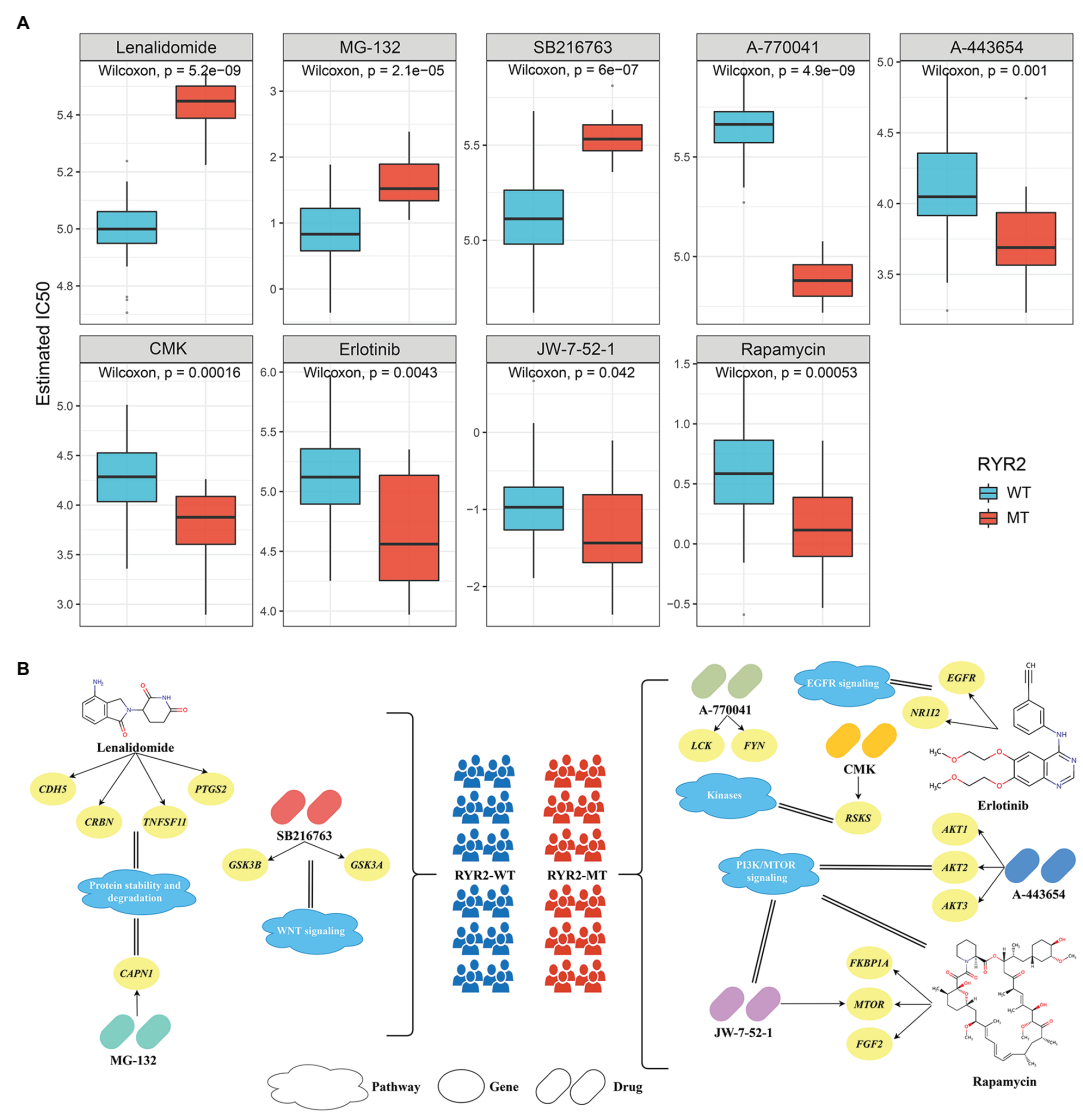
Identified potential antitumor drugs associated with *RYR2* status. **(A)** Distribution of the estimated IC50 of nine drugs between patients with and without *RYR2* mutation. **(B)** The nine drugs and their corresponding targeted molecules and pathways between patients with and without *RYR2* mutation.

We retrieved the imputed response to 138 antitumor drugs in EAC patients from a previous study to identify potential antitumor drugs with specific sensitivity to each phenotype (Geeleher et al., 2017). As shown in Figure 5A, the estimated IC50 of nine drugs significantly differed between the two groups. Patients without *RYR2* mutation were more sensitive to lenalidomide, MG-132, and SB216763, while patients with *RYR2* mutation were more sensitive to A-770041, A-443654, CMK, erlotinib, JW-7-52-1, and rapamycin. Drugs were associated with *RYR2* wild type if mainly targeting protein stability and degradation and WNT signaling, while drugs were associated with *RYR2* mutation if mainly targeting EGFR signaling, kinases, and PI3K/MTOR signaling (Figure 5B). These results provided additional reference for antitumor therapies of different *RYR2* status.

## Discussion

In the present study, we, respectively, characterized the somatic mutation landscape of 87 American EAC patients and 409 British patients from TCGA and ICGC datasets. Then, we found that *RYR2* mutated frequently in the two cohorts, and its mutation was dramatically associated with a higher TMB and a favorable prognosis. Meanwhile, patients with *RYR2* mutation suggested an “immune-hot” tumor, which enriched abundant immune-related pathways, numerous immune cell infiltrations, and higher expression of ICPs. These results indicated that patients with *RYR2* mutation might benefit more from immunotherapy, which was in line with the immunotherapy assessment results of bioinformatics algorithms.

*RYR2* is a major component of the intracellular Ca^2+^ release channels and is associated with the endoplasmic or sarcoplasmic reticulum of several cell types, especially in cardiomyocytes (Lanner et al., 2010; Van Petegem, 2015). Recent studies demonstrated that *RYR2* was significantly mutated in multiple cancers, and *RYR2* was reported to be a driver gene in cervical cancer, colon cancer, breast cancer, head and neck cancer, and lung adenocarcinoma (Wolff et al., 2018; Schmitt et al., 2019; Wang et al., 2019; Cimas et al., 2020; Wei et al., 2020). Femi (2018) demonstrated that mutation in *RYR2* was a prognosis biomarker of cervical cancer and breast cancer. Cimas et al. (2020) found that a mutation in *RYR2* was associated with a favorable outcome in basal-like breast tumors expressing *PD-1*/*PD-L1*. Wang et al. (2019) reported that *RYR2* mutation was a significant biomarker for suggesting high TMB in lung adenocarcinoma. In this study, we found that *RYR2* mutation was an independent protective prognostic factor and had a positive relationship with high TMB in EAC. TMB represents the accumulation of somatic mutations in tumors; a high TMB can give rise to mutation-derived neoantigens and improve the immunogenicity of tumor, which is likely to induce a T-cell-dependent immune response (McGranahan et al., 2016). Hence, we speculated that *RYR2* mutation might promote antitumor immunity in EAC.

Actually, the *RYR2* mutation phenotype enriched a multitude of immune-related pathways and displayed a higher abundance of immune cell infiltration, suggesting the “immune-hot” subtype. A previous study has demonstrated that the “immune-hot” tumors were more sensitive to immunotherapy (Galon and Bruni, 2019). Apart from this, concerning some prevalent biomarkers of immunotherapy such as *PD-L1*, *PD-L2*, *PD-1*, and *CTLA-4*, their expression in patients with *RYR2* mutation was higher, which was conducive to obtaining an effective immunotherapy response. Consistent with this, bioinformatics algorithms including T cell-inflamed GEP, TIDE, and SubMap methods further validated this conclusion. These results indicated that patients with *RYR2* mutation might be a promising biomarker of immunotherapy. However, the limitation of our study is in evaluating the immunotherapy response using bioinformatics algorithms rather than conducting large-scale immunotherapy clinical trials. In spite of this, the above-mentioned results were highly consistent in terms of functional analysis and predictive results, which indicates that our results are relatively reliable. In addition, we identified latent antitumor drugs associated with *RYR2* status in EAC, hoping to provide additional reference for antitumor therapies of different *RYR2* status.

In conclusion, our study identified that *RYR2* was frequently mutated in EAC, and RYR2 mutation was dramatically associated with a higher TMB and suggested a better prognosis. Moreover, *RYR2* mutation upregulated the signaling pathways implicated in immune response and enhanced the antitumor immunity in EAC. This study reveals a novel gene whose mutation could serve as a potential biomarker for the prognosis, TMB, and immunotherapy of EAC patients.

## Data Availability Statement

The original contributions presented in the study are included in the article/Supplementary Material, further inquiries can be directed to the corresponding authors.

## Author Contributions

ZLiu, YZ, and XH designed this work. ZLiu, LL, and CG integrated and analyzed the data. ZLiu, ZS, and YZ wrote the manuscript. ZLiu, DJ, ZLi, LW, and XH edited and revised the manuscript. All authors contributed to the article and approved the submitted version.

### Conflict of Interest

The authors declare that the research was conducted in the absence of any commercial or financial relationships that could be construed as a potential conflict of interest.

## Footnotes

^1^http://portal.gdc.cancer.gov/

^2^http://dcc.icgc.org/

## Supplementary Material

Supplementary Figure S1 Kaplan-Meier survival analysis of patients with gene mutations. **(A)** Kaplan-Meier survival analysis between patients with high and low tumor mutation burden. **(B)** Kaplan-Meier survival analysis between patients with and without gene mutations.

Supplementary Figure S2 Distribution of immune infiltration signatures between patients with and without *RYR2* mutation. **(A)** The infiltration abundance of 28 immune cell subsets evaluated by ssGSEA algorithm. **(B)** The immune cell infiltration pattern assessed by CIBERSORT algorithm. **(C)** The distribution of stromal, immune, and ESTIMATE scores and tumor purity between patients with and without *RYR2* mutation.
