# Supplementary figures and images for: Association of RYR2 Mutation With Tumor Mutation Burden, Prognosis, and Antitumor Immunity in Patients With Esophageal Adenocarcinoma
Source: Front Genet. 2021 May 17;12:669694. doi: 10.3389/fgene.2021.669694 (PMC8166246; doi:10.3389/fgene.2021.669694)

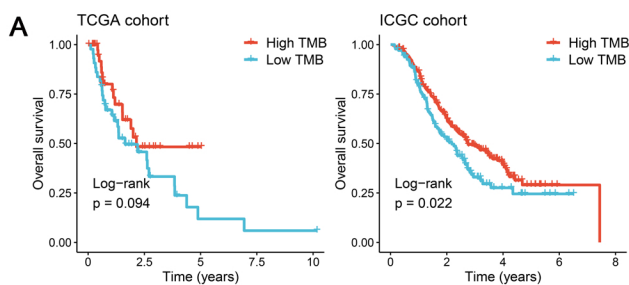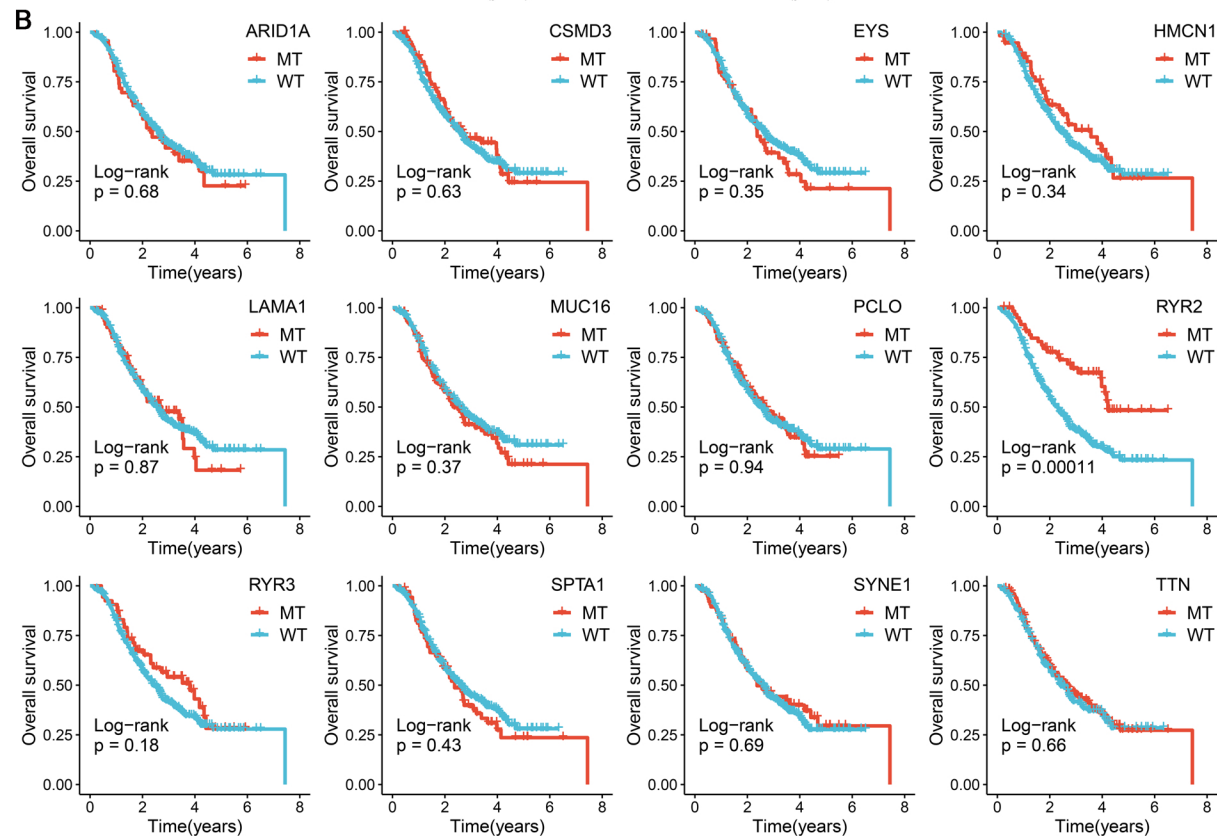

Supplement: Supplementary Figure S1 — Kaplan-Meier survival analysis of patients with gene mutations. (A) Kaplan-Meier survival analysis between patients with high and low tumor mutation burden. (B) Kaplan-Meier survival analysis between patients with and without gene mutations. [file Data_Sheet_1.PDF]

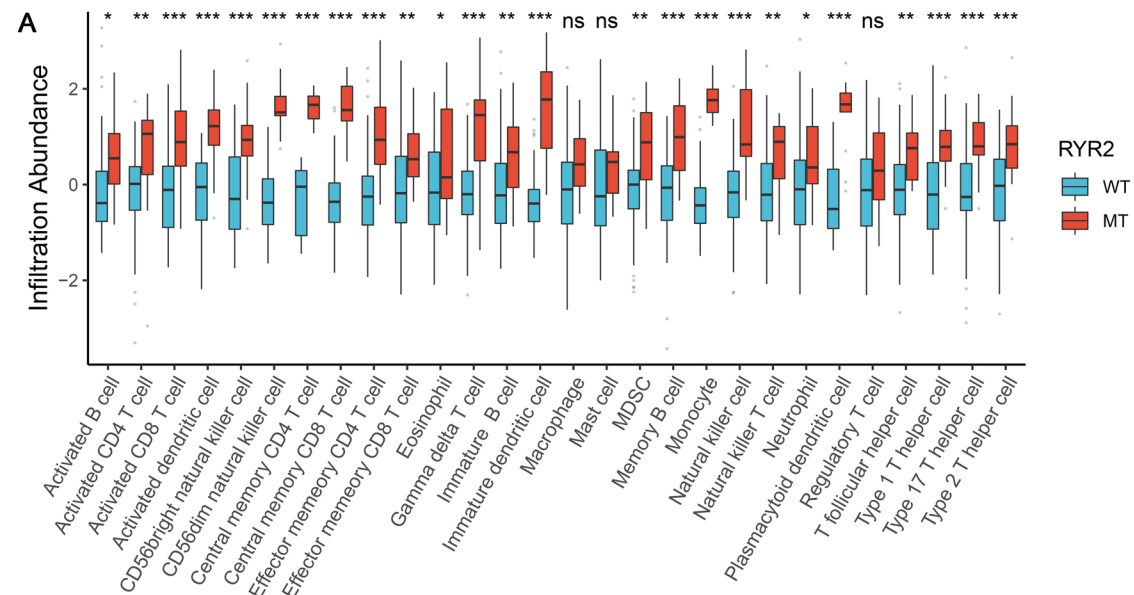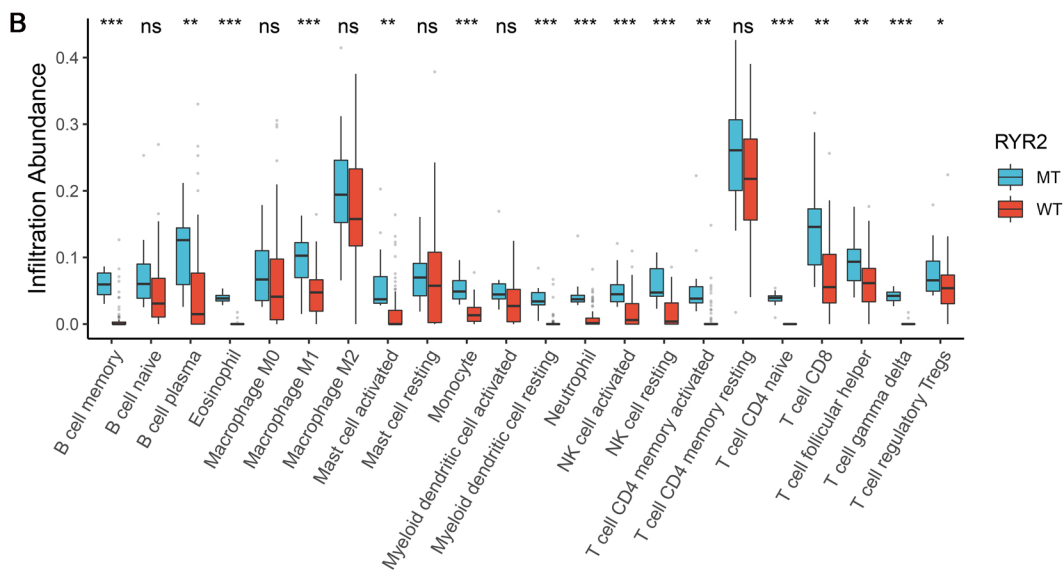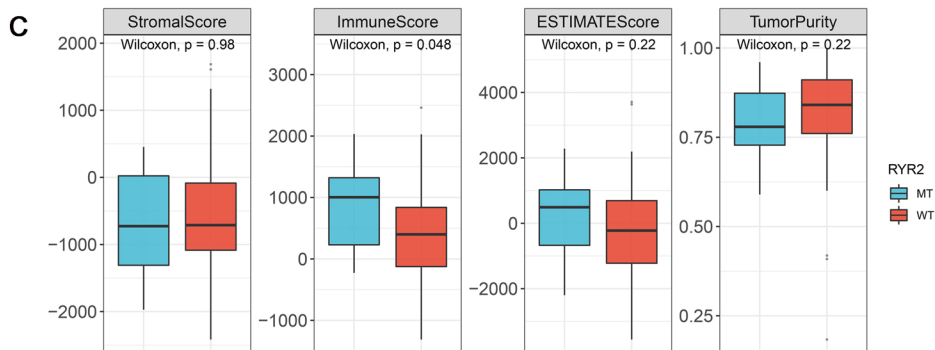

Supplement: Supplementary Figure S2 — Distribution of immune infiltration signatures between patients with and without RYR2 mutation. (A) The infiltration abundance of 28 immune cell subsets evaluated by ssGSEA algorithm. (B) The immune cell infiltration pattern assessed by CIBERSORT algorithm. (C) The distribution of stromal, immune, and ESTIMATE scores and tumor purity between patients with and without RYR2 mutation. [file Data_Sheet_2.PDF]
